# PRR7-AS1 Correlates with Immune Cell Infiltration and Is a Diagnostic and Prognostic Marker for Hepatocellular Carcinoma

**DOI:** 10.1155/2022/1939368

**Published:** 2022-08-26

**Authors:** Yifan Lu, Songhai Chen, Qingqing Wang, Jie Zhang, Xuanzeng Pei

**Affiliations:** Department of Hepatobiliary Surgery, The First Hospital of Jiaxing, Jiaxing, Zhejiang, China

## Abstract

Hepatocellular carcinoma (HCC) is a highly aggressive malignant disease, and numerous studies have shown that certain functional long noncoding RNAs (lncRNAs) are implicated in the progression of several cancers. The purpose of the research was to determine, using a database, bioinformatics, and statistical analysis, whether or not lncRNA PRR7-AS1 (PRR7-AS1) was related to HCC. TCGA datasets were used to conduct research on the PRR7-AS1 expression pattern in HCC. In order to evaluate the efficacy of GIHCG as a prognostic tool, both survival and Cox regression analyses were carried out. Furthermore, an investigation of the connection between the expression of PRR7-AS1 and immune infiltration in HCC was carried out. In this study, we identified 125 lncRNAs that were significantly dysregulated in HCC and were associated with long-term survival. Among the above 125 lncRNAs, our attention focused on PRR7-AS1. We found that PRR7-AS1 expressions were distinctly overexpressed in HCC samples compared with nontumor samples. ROC assays revealed that PRR7-AS1 effectively differentiated HCC specimens from normal tissues with an AUC of 0.875 (95% CI: 0.840 to 0.911). Moreover, the high PRR7-AS1 expression was associated with advanced clinical stage and poor prognosis of HCC patients. Importantly, the multivariate Cox proportional hazards model suggested that up-expression of PRR7-AS1 was an independent prognostic marker indicating shorter overall survival and disease-specific survival for HCC patients. Finally, we found that PRR7-AS1 expression was associated with the expression of NK CD56bright cells, Th2 cells, TFH, macrophages, Th1 cells, aDC, T helper cells, cytotoxic cells, DC, Tgd, neutrophils, and Th17 cells. Overall, the results of our study show that PRR7-AS1 was a biomarker that could be utilized to predict the prognosis of HCC patients and was linked to the infiltration of immune cells in HCC.

## 1. Introduction

Hepatocellular carcinoma (HCC) is the fifth most common cancer globally and the third most common cause of cancer-related death [1]. According to the epidemiological data, there were 841,000 newly diagnosed cases of HCC and 781,000 deaths globally; of these, China accounted for approximately half of the total number of newly diagnosed cases and fatalities [2, 3]. One of the most prominent risk factors for HCC is a chronic liver infection that is caused by hepatitis B or C virus (HBV or HCV) [4, 5]. Even though there have been significant advancements made in diagnostic modalities and standard therapies for HCC over the past two decades, recurrence and death rates are still high, with an estimated overall survival rate of roughly 12 percent after five years [6, 7]. Therefore, it is absolutely necessary to do research on the cellular process, as this will allow for the development of a diagnostic biomarker as well as a therapeutic target for the treatment of HCC.

It is now generally accepted, because to the rapid advancement of whole genome sequencing technology, transcriptome sequencing technology and the ENCODE project, that the bulk of genomic DNA is represented in processed transcripts that do not have the potential to code for protein [8]. Long noncoding RNAs (lncRNAs) are a large class of noncoding RNAs of >200 nucleotides [9]. Recent research has shown that the dysregulation of lncRNAs is linked to the development of tumors, their spread to other parts of the body, their prognosis, and their ability to be diagnosed [10, 11]. In order to control various biological processes, such as reprogramming of pluripotent stem cells, apoptosis, invasion, migration, and proliferation, they have the ability to regulate the expressions of protein-coding genes [12, 13]. However, the function of many lncRNAs has not been investigated.

The tumor microenvironment (TME), which includes stromal cells, immune cells, tumor cells, a complicated cytokine and chemokine milieu, and other components, is a dynamic system [14]. Within the TME, the immune cells and stromal cells are thought to have an essential role in the progression of various cancers [15]. According to the findings of several research studies, the stromal cells that are found within the TME are genetically stable, making them desirable therapeutic targets with a decreased likelihood of drug resistance and recurrence of the tumor [16, 17]. In addition, it is possible that a multitarget approach that simultaneously reduces TME components will give a means of treating cancer that is more successful [18, 19]. As a result, having a solid understanding of the TME is essential for preventing carcinogenesis, invasion, and metastasis, as well as for ensuring that the immune response is appropriately managed.

Recently, it was reported that lncRNA PRR7-AS1(PRR7-AS1) was significantly upregulated in colorectal cancer [20]. However, the clinical significance and prognostic value of PRR7-AS1 in HCC have not been reported previously. Thus, investigating the clinical importance of PRR7-AS1 and its correlation with TME in HCC was the purpose of our study.

## 2. Materials and Methods

### 2.1. Data Processing

The Genomic Data Commons (GDC) database (https://portal.gdc.cancer.gov/) was accessed in order to retrieve the RNA sequencing (RNA-Seq) and clinicopathological data of patients diagnosed with HCC. The investigation involved the utilization of around 374 different tumor samples.  Table 1 shows the correlation between the PRR7-AS1 expression level and clinicopathological characteristics.

### 2.2. Identification of Differentially Expressed lncRNAs

A differential analysis of the gathered information was carried out by utilizing the “limma” package of the R programming language. The criterion for screening differentially expressed long noncoding RNAs was established at a false discovery rate (FDR)-adjusted *p* value of <0.05 and a log fold change (FC) of >2. It was shown as a volcano map showing the expression of lncRNAs that were differentially expressed.

### 2.3. Univariate Cox Analysis of All lncRNAs

The clinical data that were related to the HCC samples that were found in the TCGA datasets were retrieved and applied for the assays. The information contained in the clinical data pertaining to the survival time as well as the survival status was retrieved, and the data pertaining to clinical survival as well as differential gene expression were combined. On the integrated data, a univariate Cox analysis was performed using the “survival” package in R with the default parameters. The genes with *p* values less than 0.05 were collected for further study.

### 2.4. Analysis of Immune Cell Infiltration

Using single sample gene set enrichment analysis (ssGSEA), the relative amounts of tumor infiltration caused by 24 different immune cell types were assessed [21]. The ssGSEA applies gene signatures expressed by immune cell populations and immune pathways to every cancer samples. They allowed researchers to investigate the expression levels of genes included in the published signature gene lists. The signatures that we used contained a total of 509 genes and included a wide variety of cell types involved in both the adaptive and innate immune responses. The Wilcoxon rank-sum test and Spearman correlation were utilized in order to investigate the link between PRR7-AS1 and the infiltration levels of immune cells as well as the association of infiltration of immune cells with the various expression groups of PRR7-AS1.

### 2.5. Statistical Analysis

R version 3.4.3 (RStudio, Boston, MA, USA) and GraphPad Prism version 8.01 (GraphPad Software, San Diego, CA, USA) were applied to carry out the statistical analysis. The data were expressed as mean ± SD. The Student's *t*-test or chi-square test and ANOVA test were utilized to compare the data. The Kaplan–Meier method was utilized in order to produce survival curves, and the log-rank test was utilized in order to analyze the results. For both the univariate and the multivariate regression studies, the Cox proportional hazards model was utilized. *p* < 0.05 was considered statistically significant.

## 3. Results

### 3.1. Identification of Differentially Expressed Survival-Related lncRNAs in HCC

**Figure 1:**
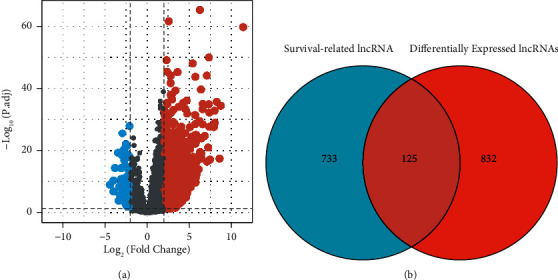
The identification of differentially expressed survival-related lncRNAs in HCC. (a) Volcano map of the differentially expressed lncRNAs in between HCC specimens and nontumor specimens. (b) Venn diagram confirmed the overlapped lncRNA between survival-related lncRNAs and differentially expressed lncRNAs.

In order to search for novel sensitive biomarkers for HCC, we evaluated TCGA datasets and found 957 lncRNAs that were expressed differently in HCC specimens compared to nontumor tissues (Figure 1(a)), which allowed us to screen for potential biomarkers. After that, we carried out univariate Cox analysis on all of the lncRNAs and discovered 858 lncRNAs that were related to survivals. Following that, we identified 125 lncRNAs that were highly dysregulated in HCC and were related with long-term survival by using the Venn analysis (Figure 1(b)).

### 3.2. The Expression of PRR7-As1 in HCC

**Figure 2:**
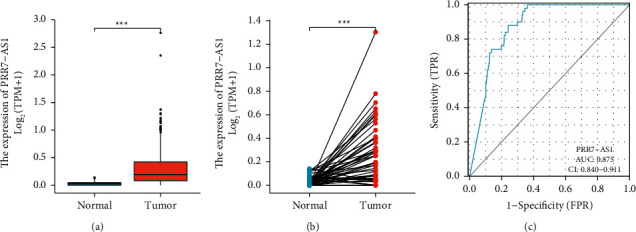
PRR7-AS1 levels in HCC specimens and its diagnostic value. (a) and (b) human PRR7-AS1 levels in HCC specimens and nontumor specimens from TCGA were determined. (c) ROC analysis of PRR7-AS1 reveals potential classification capacity between cancerous and normal tissues. ^*∗∗∗*^*p* < 0.001.

PRR7-AS1, one of the 125 lncRNAs, was the primary focus of our investigation. After searching the related studies via PubMed, we discovered that a significant number of lncRNAs had been identified. On the other hand, the function of these lncRNAs has almost never been investigated in HCC. We found that the expression of PRR7-AS1 was significantly higher in HCC specimens compared to nontumor specimens (Figures 2(a) and 2(b)). In addition, earlier research has established the diagnostic utility of lncRNAs in a variety of tumor types. As a result, we conducted additional research on the diagnostic utility of PRR7-AS1. As presented in Figure 2(c), ROC assays revealed that PRR7-AS1effectively differentiated HCC specimens from normal tissues with an AUC of 0.875 (95% CI: 0.840 to 0.911).

### 3.3. Association of PRR7-As1 Expressions with Clinical Features of HCC Patients

**Figure 3:**
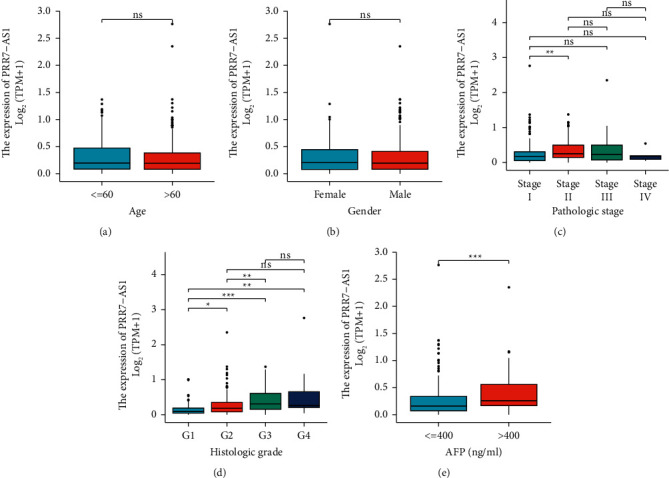
The association between the PRR7-AS1 expression and clinical factors. (a) Age. (b) Gender. (c) Pathologic stage. (d) Histologic grade. (e) AFP. ^*∗*^*p* < 0.05, ^*∗∗*^*p* < 0.01, and ^*∗∗∗*^*p* < 0.001.

Then, we explored whether PRR7-AS1 levels were correlated to clinical features of HCC cases and observed that there were no distinct differences in PRR7-AS1 expressions between HCC patients ≤ 60 and HCC patients >60 (Figure 3(a)). A similar finding was also observed between PRR7-AS1 expression and age (Figure 3(b)). However, we found that the PRR7-AS1 expression in HCC specimens with stage II was distinctly higher than that in HCC specimens with stage I (Figure 3(c)). Also, HCC specimens with advanced histologic grade and AFP>400 displayed an increased level (Figures 3(d) and 3(e)). To explore whether the overexpression of PRR7-AS1 in HCC influence the clinical progress of HCC, all cases were divided into two groups (high: *n* = 187 and low: *n* = 187) using the median expression of PRR7-AS1 as cutoff. Then, we performed chi-square test and found that the high PRR7-AS1 expression was associated with pathologic stage (*p*=0.019), AFP (*p* < 0.001), and histologic grade (*p* < 0.001) (Table 1). However, no distinct difference in the PRR7-AS1 expression was observed with other factors (all *p* > 0.05).

### 3.4. Significance of PRR7-AS1 Expression in HCC Prognosis

**Figure 4:**
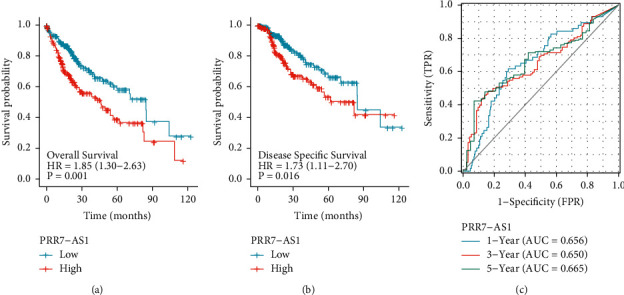
Kaplan–Meier survival curves comparing the high and low expression of PRR7-AS1 in HCC patients. (a) Overall survival. (b) Disease-specific survival. (c) The AUC for 1-, 2-, and 3-year predicted overall survival in TCGA.

To further investigate the correlation between PRR7-AS1 expressions and survival of HCC patients, our group applied the Kaplan–Meier survival and log-rank analysis. We observed that patients with the high PRR7-AS1 expression displayed a shorter overall survival (Figure 4(a), *p*=0.001) and disease specific survival (Figure 4(b), *p*=0.016) than those with a low PRR7-AS1 expression. Time-dependent ROC was carried out to examine the prognostic significance of the PRR7-AS1 expression. The AUCs for 1-, 2-, and 3-year overall survival predictions for the risk scores were 0.656, 0.650, and 0.665, respectively (Figure 4(c)). The multivariate Cox proportional hazards model suggested that up-expression of PRR7-AS1 was an independent prognostic marker indicating shorter overall survival (HR = 2.002, *p* < 0.001, Table 2) and disease specific survival (HR = 2.026, *p* < 0.005, Table 3) for HCC patients.

### 3.5. The Assays of Immune Cell Infiltration

**Figure 5:**
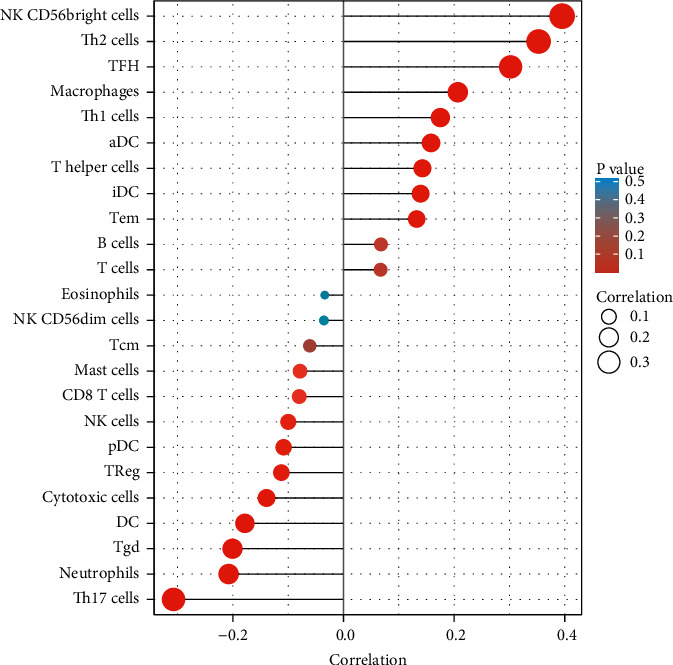
PRR7-AS1 expression levels were shown to be correlated with 24 immune cell relative abundances. Spearman's correlation coefficient values were represented by the dots' size.

To delve into the associations between PRR7-AS1 expression and immune cells, we performed ssGSEA based on TCGA datasets. We observed that PRR7-AS1 expression was associated with the expression of NK CD56bright cells, Th2 cells, TFH, macrophages, Th1 cells, aDC, T helper cells, cytotoxic cells, DC, Tgd, neutrophils, and Th17 cells(Figure 5), suggesting its function in the suppression of immunity.

## 4. Discussion

HCC is an aggressive tumor with high rates of recurrence and metastasis, which leads to the poor prognosis [22]. According to the findings of a study on the epidemiology of tumors in China, there were more than 400 thousand new and deceased patients diagnosed with HCC in 2015 [23]. Despite this, there is currently no viable diagnosis or treatment strategy for this disease. At the moment, AFP is the most important clinical diagnostic measure for HCC; however, recent research has shown that AFP expression can also increase in other liver illnesses, such as hepatitis; as a result, its specificity is quite poor [24, 25]. Consequently, the solution to this issue lies in the discovery of biological indices that have a high level of both sensitivity and specificity. Previous research has shown that lncRNAs were detectable and also persistent in the serum of cancer patients, which paved the way for them to be used as diagnostic biomarkers for a variety of malignancies [26, 27]. However, the therapeutic application of circulating lncRNAs for the treatment of HCC is still a contentious topic due to the conflicting outcomes reported by different groups as well as some technical problems.

In this research, we analyzed TCGA datasets and found 125 differentially expressed survival-related lncRNAs in HCC. Then, we checked the 125 lncRNAs in “PubMed,” and found many of them have been functionally reported in HCC. However, PRR7-AS1, as a newly identified lncRNA, has not been investigated in HCC. To date, only a study reported that the PRR7-AS1 expression was distinctly increased in colorectal cancer specimens. In addition, its prognostic value was also demonstrated [20]. In this study, we first reported that PRR7-AS1 expression was distinctly increased in HCC specimens compared with nontumor specimens. ROC assays revealed that PRR7-AS1effectively differentiated HCC samples from normal samples with AUC of 0.875 (95% CI: 0.840 to 0.911), highlighting its potential used as a diagnostic factor for HCC. Moreover, we confirmed that high PRR7-AS1 expression was associated with advanced clinical stage and poor prognosis of HCC patients. More importantly, the multivariate Cox proportional hazards model suggested that the up-expression of PRR7-AS1 was an independent prognostic marker indicating shorter overall survival and disease-specific survival for HCC patients. Our finding provided a basis for the application of PRR7-AS1 as a novel diagnostic and prognostic biomarker of HCC.

Immunotherapies have become more widely used in the treatment of HCC patients in recent years, which has resulted in a shift in the overall therapy paradigm for HCC [28]. It is common knowledge that tumor-infiltrating immune cells(TIICs) have an effect on the body's immune response [29, 30]. TIIC process abnormal patterns of biological behavior in a convoluted fashion and exert an essential action in response to immunotherapies. In addition, the critical genes that are connected with immunological components of the TME are very useful as prognostic indicators. It has been established that characteristics of the tumor microenvironment can act as biomarkers to evaluate the responses of tumor cells to immunotherapy and to influence prognostic outcomes. Another important discovery from this study was the correlation between the level of CDT1 mRNA and the degree of immune cell infiltration in HCC [31, 32]. CDT1 expression was distinctly related to the abundance of Th2 cells, mast cells, cytotoxic cells, dendritic cells, neutrophils, *T* follicular helper cells, and activated dendritic cells. Th cells are essential immune regulatory cells found in the body, and the ratio of Th1 cells to Th2 cells is normally maintained in a state of dynamic equilibrium [33, 34]. We found that the PRR7-AS1 expression was associated with the expression of NK CD56bright cells, Th2 cells, TFH, macrophages, Th1 cells, aDC, *T* helper cells, cytotoxic cells, DC, Tgd, neutrophils, and Th17 cells, suggesting its function in the suppression of immunity.

There are several limitations in the current research. First, there was no experimental validation for our findings and RT-PCR or high-throughput sequencing was needed to further demonstrate our results. Second, PRR7-AS1 has a high expression level and is associated with a poor prognosis in HCC; however, the particular mechanism behind this association has not been validated. Therefore, clarifying the correctness of an integrated study of PRR7-AS1 expression and establishing the particular link of PRR7-AS1 with tumor immunosuppressive microenvironment should be the primary focuses of future research. More in vivo and in vitro studies are needed.

## 5. Conclusion

The current study provided evidence that PRR7-AS1 contributed to a worsening of the TME and was substantially expressed in HCC. An increased level of PRR7-AS1 expression was found to be associated with lower levels of a variety of TIICs, making it an independent prognostic factor. Our research has provided new insights into the inhibitory function of PRR7-AS1 in the TME and the possibility of PRR7-AS1 as a diagnostic and prognostic biomarker in HCC.

## Figures and Tables

**Table 1:** Correlation between the PRR7-AS1 expression level and clinicopathological characteristics.

Characteristics	Low expression of PRR7-AS1	High expression of PRR7-AS1	*p*
*n*	187	187	
*Pathologic stage ( n (%))*			0.019
Stage I	98 (28%)	75 (21.4%)	
Stage II	32 (9.1%)	55 (15.7%)	
Stage III	41 (11.7%)	44 (12.6%)	
Stage IV	3 (0.9%)	2 (0.6%)	
*Gender (n (%))*			1.000
Female	60 (16%)	61 (16.3%)	
Male	127 (34%)	126 (33.7%)	
*Age (n (%))*			0.797
≤60	87 (23.3%)	90 (24.1%)	
>60	100 (26.8%)	96 (25.7%)	
*AFP (ng/ml) (n (%))*			<0.001
≤400	125 (44.6%)	90 (32.1%)	
>400	19 (6.8%)	46 (16.4%)	
*Histologic grade (n (%))*			<0.001
G1	42 (11.4%)	13 (3.5%)	
G2	97 (26.3%)	81 (22%)	
G3	43 (11.7%)	81 (22%)	
G4	3 (0.8%)	9 (2.4%)	
Age, median (IQR)	61 (54, 69)	61 (51, 69)	0.381

**Table 2:** Univariate and multivariate analyses for overall survival in HCC patients.

Characteristics	Total (N)	*Univariate analysis*		*Multivariate analysis*	
		Hazard ratio (95% CI)	*p* value	Hazard ratio (95% CI)	*p* value
*Gender*	373				
Female	121	Reference			
Male	252	0.793 (0.557–1.130)	0.200		
*Age*	373				
≤60	177	Reference			
>60	196	1.205 (0.850–1.708)	0.295		
*Histologic grade*	368				
G1 & G2	233	Reference			
G3 & G4	135	1.091 (0.761–1.564)	0.636		
*Pathologic stage*	349				
Stage I and Stage II	259	Reference			
Stage III and Stage IV	90	2.504 (1.727–3.631)	**<0.001**	2.651 (1.825–3.852)	**<0.001**
*PRR7-AS1*	373				
Low	187	Reference			
High	186	1.850 (1.301–2.629)	**<0.001**	2.002 (1.374–2.916)	**<0.001**

**Table 3:** Univariate and multivariate analyses for disease-specific survival in HCC patients.

Characteristics	Total (N)	*Univariate analysis*		*Multivariate analysis*	
		Hazard ratio (95% CI)	*p* value	Hazard ratio (95% CI)	*p* value
*Gender*	365				
Female	118	Reference			
Male	247	0.813 (0.516–1.281)	0.373		
*Age*	365				
≤60	174	Reference			
>60	191	0.846 (0.543–1.317)	0.458		
*Histologic grade*	360				
G1 & G2	227	Reference			
G3 & G4	133	1.086 (0.683–1.728)	0.726		
*Pathologic stage*	341				
Stage I and Stage II	254	Reference			
Stage III and Stage IV	87	3.803 (2.342–6.176)	**<0.001**	4.095 (2.512–6.674)	**<0.001**
*PRR7-AS1*	365				
Low	186	Reference			
High	179	1.727 (1.105–2.698)	**0.016**	2.026 (1.233–3.331)	**0.005**

## Data Availability

The datasets used and/or analyzed in this study are available from the corresponding author upon reasonable request.
